# Impact of Regional Versus General Anaesthesia on Outcomes Following Total Knee Replacement: A Systematic Review and Meta-Analysis

**DOI:** 10.7759/cureus.95445

**Published:** 2025-10-26

**Authors:** Abdelfatah M Elsenosy, Eslam Hassan, Ahmed S Yousef, Mustafa Al-Alawi, Wael Elbagory

**Affiliations:** 1 Trauma and Orthopaedics, University Hospital Dorset, Poole, GBR; 2 Trauma and Orthopaedics, Poole General Hospital, Poole, GBR; 3 Anaesthesia, Southend University Hospital, Southend-on-Sea, GBR

**Keywords:** blood transfusion, general anaesthesia, length of stay, meta-analysis, postoperative complications, regional anaesthesia, systematic review, total knee replacement

## Abstract

Total knee replacement (TKR) is widely performed for advanced osteoarthritis, and the choice between regional anaesthesia (RA) and general anaesthesia (GA) may influence perioperative outcomes. This systematic review and meta-analysis aimed to compare the impact of RA versus GA on outcomes following TKR. A comprehensive search of PubMed, Embase, Scopus, Web of Science, and the Cochrane Library was conducted for comparative studies published between January 2010 and May 2025. Nine retrospective cohort studies encompassing 522,080 TKR procedures were included. RA was associated with significantly lower odds of blood transfusion (odds ratio (OR): 0.58; 95% confidence interval (CI): 0.44-0.77; p<0.001), fewer 30-day postoperative complications (OR: 0.59; 95% CI: 0.39-0.88; p=0.01), and a shorter hospital stay (standardised mean difference (SMD): -0.09; 95% CI: -0.14 to -0.04; p<0.001). Despite moderate-to-high heterogeneity, the direction of effect consistently favoured RA. These findings suggest that RA provides superior short-term outcomes compared with GA in TKR, supporting its preferential use, particularly in high-risk or elderly patients, while reinforcing the importance of individualised anaesthetic planning.

## Introduction and background

Knee osteoarthritis (KOA) is a major global health problem, contributing substantially to disability, healthcare costs, and reduced quality of life. Its prevalence has risen steadily due to ageing populations, obesity, and sedentary lifestyles [1]. According to the Global Burden of Disease (GBD) study, the global age-standardised prevalence of KOA increased by 7.5%, and the years lived with disability (YLD) rate increased by 7.8% between 1990 and 2019, with women disproportionately affected and disease burden rising sharply with age [2].

Regional trends mirror these findings. In the Middle East and North Africa (MENA), KOA prevalence more than doubled between 1990 and 2019, reaching 17.75 million cases, with women showing higher YLD rates [3]. In China, KOA-related disability remains high among older adults and rural populations, with a national burden exceeding four million YLDs [4]. Similar patterns are seen in Kazakhstan, where admission rates among women are three times higher than men, underscoring regional disparities and the need for targeted prevention [5].

Total knee replacement (TKR) remains the established intervention for advanced KOA with disabling pain, limited mobility, and radiographic progression despite conservative management [6,7]. Advances in implant design, surgical techniques, and perioperative protocols have improved functional outcomes and recovery. Modern implants enhance congruence and kinematics, while minimally invasive and computer-assisted surgery (CAS) reduces blood loss and hospital stay by up to 25-30% and 1-2 days, respectively [8-10]. Enhanced Recovery After Surgery (ERAS) and fast-track pathways integrate multimodal analgesia, early mobilisation, and patient education, further shortening hospital stay and reducing complications [11-15].

Anaesthetic choice is central to perioperative optimisation. Regional anaesthesia (RA), including neuraxial (spinal or epidural) and motor-sparing peripheral techniques such as adductor canal and iPACK blocks, is increasingly favoured over general anaesthesia (GA) due to superior analgesia, lower opioid requirements, earlier mobilisation, and better integration with ERAS protocols [16,17]. Large registry data support RA, linking it to lower complication rates, shorter hospitalisation, and fewer readmissions compared to GA [18].

However, RA's advantages are not universal. Some studies report improved early pain control without significant reduction in long-term pain [19], while others show higher home discharge rates and reduced readmissions [20]. Combined GA and RA may optimise satisfaction and analgesia [21], though RA carries risks such as transient neurological effects and anxiety if poorly explained [22]. Importantly, RA has been associated with lower cardiovascular and renal complications and reduced ICU admissions compared to GA, suggesting systemic benefits [23]. Therefore, the primary objective of this systematic review and meta-analysis was to compare perioperative outcomes, including blood transfusion rates, postoperative complications, and hospital length of stay, between RA and GA in TKR. The secondary objective was to evaluate organ-specific complications, readmission rates, and mortality where data were available.

## Review

This systematic review and meta-analysis aimed to compare RA and GA in TKR by examining differences in perioperative blood transfusion, complication rates, and hospital length of stay.

Methods

Search Strategy

A comprehensive literature search was performed in PubMed, Embase, Scopus, Web of Science, and the Cochrane Library for studies published between January 2010 and May 2025. The search included both Medical Subject Headings (MeSH) and free-text terms to capture all relevant comparative studies evaluating RA versus GA in TKR. The following search terms were applied: (“total knee arthroplasty” OR “total knee replacement” OR TKA OR TKR) AND (“regional anaesthesia” OR spinal OR epidural OR neuraxial) AND (“general anaesthesia” OR GA). Equivalent adaptations of this strategy were applied to each database. All retrieved citations were imported into EndNote reference manager (Clarivate, London, United Kingdom), where duplicate records were automatically detected and removed, followed by manual verification to ensure accuracy before screening. Reference lists of the included studies and relevant reviews were manually searched to identify any additional eligible publications. ClinicalTrials.gov was also screened for unpublished or ongoing studies.

The search was limited to human studies and English-language articles published within peer-reviewed journals. No randomised controlled trials (RCTs) were identified; all included studies were retrospective cohort analyses. This was anticipated a priori, as the available evidence in this field is largely derived from large-scale registry and database cohorts rather than experimental trials.

Inclusion and Exclusion Criteria

The final inclusion and exclusion criteria used for study selection are outlined in Table 1.

**Table 1:** Summary of the inclusion and exclusion criteria used for study selection in this systematic review and meta-analysis PICO: Population, Intervention, Comparison, Outcomes; RA: regional anaesthesia; GA: general anaesthesia

PICO element	Inclusion criteria	Exclusion criteria
Population	Adults (≥18 years) undergoing total knee replacement, including primary, bilateral, ambulatory, or revision procedures	Paediatric populations
Intervention	RA, including neuraxial techniques (spinal, epidural, or combined spinal-epidural) with or without adjunctive peripheral nerve blocks	Studies focused solely on peripheral nerve blocks without a neuraxial component
Comparator	GA delivered using volatile or intravenous agents, with or without supplementary regional blocks	Studies lacking a GA comparator group
Outcomes	Quantifiable perioperative outcomes such as blood transfusion rate, hospital length of stay, overall or organ-specific complications, readmission, reoperation, or mortality	Studies not reporting measurable perioperative outcomes
Study design and period	Comparative studies (randomised or observational) directly evaluating RA versus GA in total knee replacement, published in English-language peer-reviewed journals between January 2010 and May 2025	Case reports, review articles, editorials, conference abstracts, cadaveric or biomechanical studies, and duplicate publications based on the same dataset (most comprehensive or recent version retained)

Outcome Measures

Primary outcomes were perioperative allogeneic blood transfusion requirement, overall 30-day complication rate, and postoperative hospital length of stay. 

Secondary outcomes, analysed when data were available, included organ-specific complications (e.g., cardiovascular, thromboembolic, renal, respiratory, surgical site infection), 30-day readmission, reoperation, or revision, patient-reported outcome measures, and all-cause mortality.

Data Extraction and Quality Assessment

Reviewers independently extracted study characteristics (author, year, design, sample size, demographic profile, anaesthetic details, follow-up duration) and numerical outcome data. Discrepancies were resolved by discussion and, when necessary, by a third reviewer.

Risk of bias for non-randomised studies was assessed using the Risk of Bias in Non-randomised Studies of Interventions (ROBINS-I) tool. This framework evaluates seven domains: confounding, participant selection, intervention classification, deviations from intended interventions, missing data, outcome measurement, and selective reporting. Each domain was scored independently before assigning an overall judgement of low, moderate, serious, or critical risk of bias. Any disagreements in scoring were resolved by consensus, ensuring consistent application of the ROBINS-I criteria.

Statistical Analysis

Meta-analyses were conducted using Review Manager (RevMan) Version 5.4 (The Cochrane Collaboration, London, England, United Kingdom). Dichotomous outcomes were pooled as odds ratios (ORs) with 95% confidence intervals (CIs), while continuous outcomes were summarised as standardised mean differences (SMDs) with 95% CIs.

A random-effects model (DerSimonian-Laird) was used by default to account for anticipated clinical and methodological heterogeneity; a fixed-effect model was applied only when heterogeneity was negligible. Between-study heterogeneity was quantified using the chi-squared (Q) test and I² statistic, with I² >50% interpreted as moderate-to-high heterogeneity.

Publication bias was assessed visually using funnel plots and formally with Egger's regression test (two-tailed significance threshold p<0.05). All statistical tests were two-sided, and results were considered statistically significant at p<0.05.

Results

Search and Study Selection

**Figure 1:**
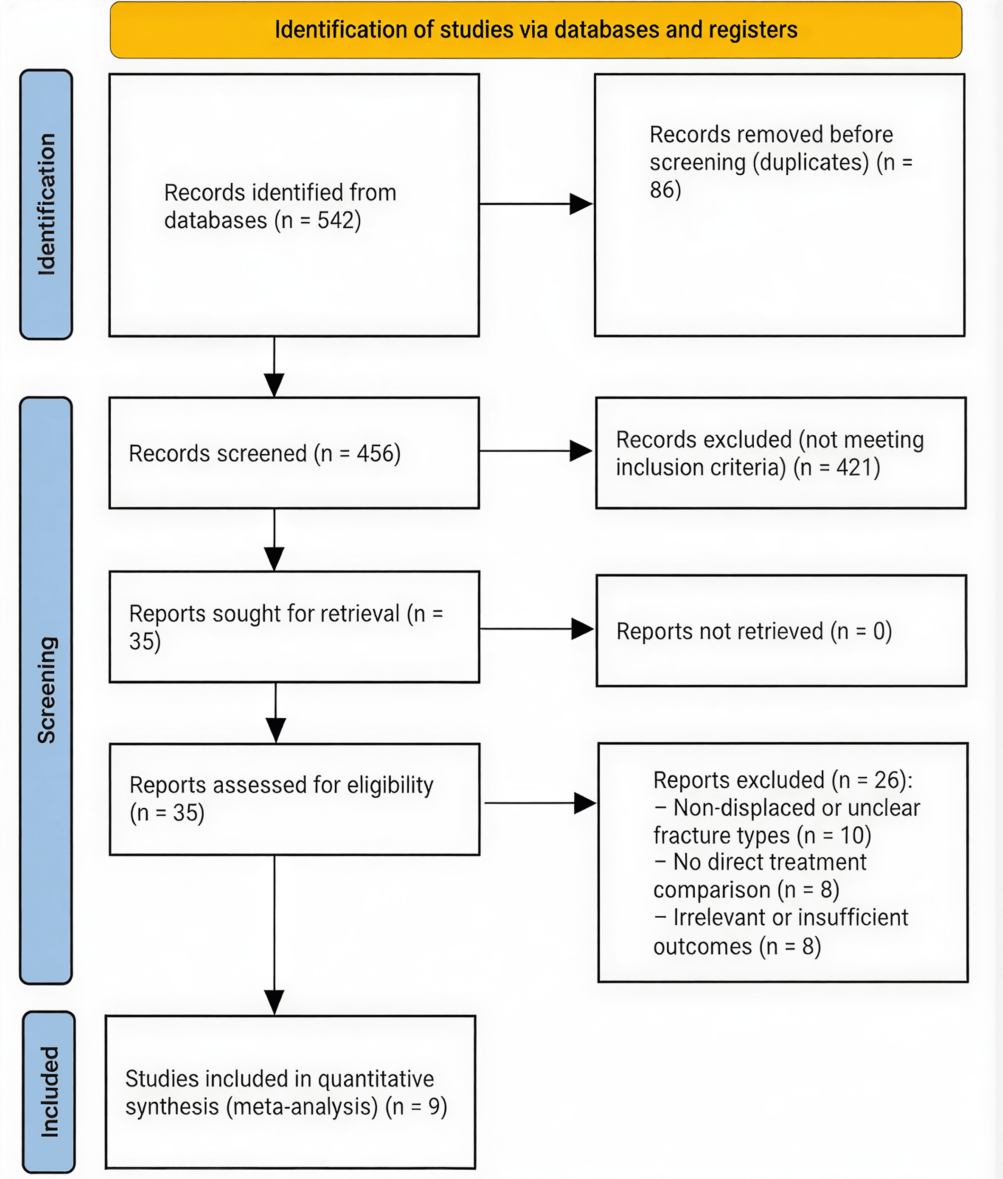
PRISMA flowchart for the included studies PRISMA: Preferred Reporting Items for Systematic Reviews and Meta-Analyses

A systematic search of PubMed, Embase, Scopus, Web of Science, and the Cochrane Library (January 2010 to May 2025) identified a total of 542 records, distributed as follows: PubMed=172, Embase=136, Scopus=104, Web of Science=89, and Cochrane Library=41. An additional 10 records were identified through manual screening of reference lists. After removing 86 duplicates using EndNote's automated and manual verification processes, 456 titles and abstracts were screened, of which 421 were excluded for irrelevance. Thirty-five full-text articles were reviewed in detail, and 26 were excluded due to non-comparative design, inadequate outcome data, or overlapping cohorts. Ultimately, nine retrospective cohort studies met all inclusion criteria and were included in the quantitative synthesis. No RCTs were identified, indicating that current evidence regarding anaesthetic choice in TKR remains limited to observational data. The study selection process, including the number of records retrieved from each database, is summarised in the Preferred Reporting Items for Systematic Reviews and Meta-Analyses (PRISMA) flow diagram (Figure 1).

Study Characteristics

This meta-analysis included nine retrospective cohort studies comparing RA with GA for TKR, encompassing a total of 522,080 procedures: 333,336 under RA and 188,744 under GA. The studies evaluated primary unilateral TKR, simultaneous bilateral TKR, ambulatory (outpatient) TKR, and aseptic revision TKR. All studies provided level III evidence, with eight employing propensity score matching or multivariable adjustment to minimise confounding.

Patients were typically older adults (mean or median age: 64-70 years) with an overweight body mass index (BMI) (≈26-34 kg/m²). Civilian cohorts were predominantly female, while the veteran cohort was almost exclusively male. RA protocols consisted mainly of single-shot spinal, epidural, or combined spinal-epidural blocks, often supplemented with light intravenous sedation or peripheral nerve blocks. Two studies included a combined GA+RA arm, which was analysed separately from pure GA. Follow-up was short-term, limited to index admission, 72 hours, 30 days, or 90 days, reflecting the database capture windows.

Primary outcomes encompassed hospital length of stay, perioperative room times, composite and organ-specific complications (including transfusion, wound infection, cardiovascular, respiratory, thromboembolic, renal, and urinary events), readmission, reoperation, or revision, patient-reported outcomes, and mortality. Across studies, RA was consistently associated with clinically meaningful advantages, namely, 0.3-0.5 days shorter hospital stay, 10-18% relative reductions in overall complications, and 25-50% lower odds of transfusion or cardiorenal events, without increases in venous thromboembolism or mortality.

No study reported disadvantages associated with RA. Several authors recommended RA as the preferred anaesthetic technique, particularly for high-risk, bilateral, or ambulatory TKR cases. 

Key characteristics of the included studies, including design, patient demographics, interventions, follow-up periods, outcome measures, and main findings, are summarised in Table 2.

**Table 2:** Summary of study characteristics, including sample sizes, patient demographics, anaesthetic interventions, follow-up periods, complication profiles, and primary outcomes, for the nine retrospective cohort studies comparing RA with GA in TKR (primary unilateral, simultaneous bilateral, ambulatory, and aseptic revision procedures) TKR: total knee replacement; RA: regional anaesthesia; GA: general anaesthesia; BMI: body mass index; PROMs: patient-reported outcome measures; EQ-5D: EuroQol-5 Dimension; OKS: Oxford Knee Score; LOS: length of stay; OR: odds ratio; CI: confidence interval; SSI: surgical site infection; VTE: venous thromboembolism; PE: pulmonary embolism; BTKA: bilateral total knee arthroplasty; THR: total hip replacement; NHIS: National Health Interview Survey; PSM: propensity score matching; IQR: interquartile range; ICU: intensive care unit; MI: myocardial infarction; ARF: acute renal failure; ACS-NSQIP: American College of Surgeons National Surgical Quality Improvement Program; UTI: urinary tract infection; SA: spinal anaesthesia; CCI: Charlson Comorbidity Index; ASA: American Society of Anesthesiologists; DVT: deep-vein thrombosis; NA: neuraxial anaesthesia; Aes: adverse events; SAE: serious adverse events; US VASQIP: United States Veterans Affairs Surgical Quality Improvement Program; AOR: adjusted odds ratio

Study	Study design	Sample size	Level of evidence	Patient demographics	Intervention details	Follow-up	Outcome measures	Results	Complications	Conclusions
Matharu et al. [18]	National Joint Registry➔Hospital Episode Statistics, retrospective registry cohort (primary TKR)	426,104 TKR patients (RA=298,015; GA=128,089)	III	Mean age 70.2±9.2 years; 57% female; mean BMI 31.1±5.5 kg/m² (regional and general groups balanced)	RA (spinal±sedation/nerve block) vs. GA (±nerve block)	90 days post-surgery	Length of stay, readmission, any and specific complications, re-operation, revision, mortality, PROMs (EQ-5D, OKS)	RA decreased LOS by 0.47 days (β=-0.47; p<0.001) and reduced readmission (OR 0.91) and any complication (OR 0.90) vs. GA	Lower risks with RA: UTI (OR 0.87), SSI (OR 0.84), and anaemia (OR 0.89); no significant difference in VTE, PE, or mortality	RA yielded shorter stays and fewer complications; authors recommend it as the reference standard technique for TKR (and THR)
Lee et al. [23]	Korean NHIS sample cohort, 1:1 PSM (primary TKR)	6,491 primary TKRs identified (GA=943; RA=5,548); after PSM 1,886 patients (943 vs. 943)	III	Median age 70 years (IQR 64-74); ~13% male; median BMI 25.8 kg/m²; median Elixhauser score 7 (balanced post-match)	GA (volatile/IV) vs. RA (spinal, epidural, or combined spinal-epidural)	30 days post-surgery	30-day mortality, transfusion, length of stay, total cost, ICU admission, broad complication set (MI, ARF, stroke, VTE, SSI, etc.)	No difference in mortality (0.32% vs. 0%), transfusion (84.5% vs. 84.7%), or LOS (median 50 vs. 53 days); GA incurred higher total cost (₩ 8.07 M vs. 7.49 M; p=0.0002)	GA ↑ ICU admission (8.48% vs. 2.33%), MI (1.7% vs. 0.64%), ARF (0.85% vs. 0.11%)	RA lowers major cardiorenal events, ICU use, and costs without affecting mortality or stay; authors favour RA for TKR, especially in high-risk patients
Pugely et al. [24]	ACS-NSQIP 2005-2010, retrospective cohort (primary TKR)	14,052 TKAs: 6,030 SA (42.9%) vs. 8,022 GA (57.1%)	III	Mean age ≈67 years; ~64% female; BMI ≈33 kg m⁻²; predominantly White (≈79-81%); comorbidity profile balanced after propensity matching	GA (volatile/intravenous) vs. single-shot SA; other anaesthetic types excluded	30-day postoperative period	Primary: any 30-day complication. Secondary: specific complications (e.g., wound infection, transfusion), operative time, LOS, 30-day mortality. Multivariate logistic and propensity score analyses	Overall complication rate lower with SA (10.72%) vs. GA (12.34%; p=0.003). GA independently increased risk of any complication (OR 1.129; 95% CI 1.004-1.269). The spinal group had fewer superficial wound infections (0.68% vs. 0.92%) and transfusions (5.02% vs. 6.07%), shorter surgery (96 vs. 100 min), and hospital stay (3.45 vs. 3.77 days; all p<0.001)	Types and rates reported (see Results); no 30-day mortality difference; GA associated with higher superficial infection, transfusion, and overall complications	GA confers a small but significant rise in 30-day morbidity versus SA. Absolute differences are modest, but benefit of SA greatest in patients with multiple comorbidities; SA should be considered preferentially in high-risk TKA patients
Wei et al. [25]	ACS-NSQIP 2014-2017, retrospective cohort (aseptic revision TKR)	8,820 revision TKAs (GA=3,192; RA=3,474; GA+RA=2,154)	III	Mean age 65±10 years; 62% female; mean BMI 33.6±7.2 kg m⁻²; 78% White; baseline comorbidities balanced after adjustment	GA (volatile/IV) vs. RA (spinal/epidural) vs. combined GA+RA	30 days post-surgery	Any 30-day complication, perioperative blood transfusion, extended LOS >7 days, specific wound/pulmonary/renal/UTI/VTE/cardiac events, mortality, re-operation	RA vs. GA: lower odds of any complication (OR 0.78, p = 0.008); transfusion (OR 0.55, p < 0.001); extended LOS (OR 0.46, p < 0.001)	No adjusted differences in wound, pulmonary, renal, UTI, VTE, cardiac, or septic complications or mortality	RA reduced overall morbidity, transfusion need, and LOS vs. GA in revision TKA; the authors suggest RA (or combined) as the preferred technique pending prospective confirmation
Park et al. [26]	Single-centre retrospective cohort 2005-2014 (primary unilateral TKR)	1,236 TKAs (GA=490; SA=746)	III	Mean age 69.8±7.5 years; 89.9% female; mean BMI 26.6 kg/m²; modified CCI 3.1±1.5 (slightly higher in SA)	GA (nitrous oxide+oxygen+desflurane±fentanyl) vs. SA with 8-12 mg 0.5% hyperbaric bupivacaine+propofol sedation; identical surgical and rehab protocols	30 days post-surgery (mean 29.7±3.1 days)	OR pre-/post-room times, operative time, LOS, transfusion, any and specific 30-day complications, mortality	GA vs. SA: +9.4 min pre-op room time, +12.7 min post-op room time, +2.5 days hospital stay (all p≤0.001); operative time similar	GA had higher transfusion (41.8% vs. 35.1%; OR 1.08; p=0.01) and SSI (1% vs. 0%; p=0.005); any adverse event 45.1% vs. 37.5% (OR 1.09); no differences in VTE, MI, stroke, mortality	GA is linked to longer perioperative times, longer stay, and modestly higher SSI and transfusion rates; the authors advise favouring SA when feasible
Walker et al. [27]	ACS-NSQIP 2007-2013, 2:1 PSM (simultaneous bilateral TKR)	1,957 bilateral TKAs after exclusions (GA=1,435; NA=522); matched cohorts: 1,566 (GA=1,044; NA=522)	III	Median age 64 years; 44% male; median BMI 32.7 kg m⁻²; ASA I-III; comorbidities balanced post-match	NA (spinal, epidural, or combined) vs. GA during simultaneous bilateral TKA	30 days post-surgery	Transfusion rate and units, hospital LOS, superficial/deep SSI, wound dehiscence, pneumonia, re-intubation, PE, DVT, renal injury, UTI	NA reduced transfusion need (26% vs. 40%; p<0.0001) and units (0.26±0.45 vs. 0.41±0.49; p<0.0001); LOS similar (3.67±1.92 vs. 3.52±1.61 days; p=0.14)	No significant differences in PE, DVT, SSI, pneumonia, renal injury, or UTI between groups	NA lowers blood-product transfusion requirements without affecting other 30-day outcomes; the authors advocate NA as preferable in bilateral TKA
Stundner et al. [28]	Premier Perspective 2006-2010, retrospective cohort (simultaneous bilateral TKR)	15,687 BTKAs: NA=1,066; GA=12,567; combined GA+NA=2,054	III	Mean age 63.9 years (NA) vs. 64.6 years (GA); 58% female overall; comorbidity burden similar across groups	NA (spinal/epidural) vs. GA (inhalational/IV) vs. combined GA+NA during elective simultaneous BTKA	In-hospital and 30-day postoperative period	In-hospital and 30-day mortality, composite major complications, blood transfusion, mechanical ventilation, median LOS	NA lowered transfusion need 28.5% vs. 44.7% (OR 0.52; p<0.0001); median LOS 2.8 days vs. 3.1 days (GA) (p<0.0001); 16% adjusted reduction in major complications (NS); mortality similar	No significant differences in individual major complications; clear transfusion advantage with NA; other outcomes trended in NA's favour but did not reach significance	NA (alone or with GA) significantly reduces transfusion requirements and shows a trend toward lower morbidity; the authors advocate NA as part of a multimodal strategy to improve BTKA outcomes
Kendall et al. [29]	ACS-NSQIP 2011-2018, 1:1 PSM (ambulatory/out-patient TKR)	5,574 out-patient TKAs (GA=2,034; SA=3,540); matched cohort 3,924 (1,962 vs. 1,962)	III	Mean age 64.6±10.2 years (GA) vs. 64.4±8.7 years (SA); 46% male; mean BMI 32.4 kg m⁻²; ASA I-II 58%	GA (volatile/IV) vs. single-shot SA during ambulatory TKA	72 hours post-surgery (early Aes); 30-day readmission captured	Composite SAE, minor Aes, any Aes, blood transfusion, readmission, failure-to-rescue	SAE no difference (0.92% GA vs. 0.66% SA; p=0.369). GA ↑ minor Aes (2.09% vs. 0.51%), any Aes (2.91% vs. 1.02%), and transfusion need (1.68% vs. 0.41%; p<0.001)	Excess GA complications driven by transfusion and pneumonia; no differences in VTE, cardiac, renal events, readmission, or failure-to-rescue	SA lowers minor morbidity and transfusion without affecting serious Aes; the authors advise favouring SA for ambulatory TKR when feasible
Baldawi et al. [30]	US VASQIP 2008-2015 veterans, 1:1 PSM (primary TKR)	48,282 primary TKAs: GA 32,363 vs. NA 14,395; matched 14,395+14,395	III	Veterans; 51% ≥65 years (NA) vs. 44% (GA), 94% male overall; BMI >30 kg m⁻² in ≈59%; ASA III-IV ≈73%	GA (volatile/IV) vs. NA (spinal/epidural/regional)	30 days post-op	30-day mortality; cardiovascular, respiratory, and renal composites; DVT/PE; SSI; mean and prolonged LOS	After matching, NA vs. GA: cardiovascular AOR 0.74; respiratory 0.75; renal 0.62; DVT 0.76; pneumonia 0.70; mean LOS 4.9 vs. 5.2 days (p<0.001); prolonged LOS AOR 0.85	No difference in mortality (0.25% vs. 0.27%) or SSI (0.95% vs. 1%); NA lowered deep-organ SSI trend but NS	NA reduced cardiorespiratory-renal morbidity and hospital stay; the authors recommend NA when feasible in veteran TKA patients

Quality Assessment of the Included Studies: Risk of Bias

The risk of bias in the included non-randomised studies was evaluated using the ROBINS-I tool. Eight of the nine cohort studies were rated as having a moderate overall risk of bias, primarily due to potential residual confounding that could not be completely addressed despite multivariable adjustment or propensity score matching. These eight studies were judged to have low risk of bias in all other domains, including participant selection, intervention classification, deviations from intended interventions, missing data, outcome measurement, and selective reporting.

The single-centre study by Park et al. carried a serious risk of bias due to residual imbalances in American Society of Anesthesiologists (ASA) class, BMI, and sedation depth. No study was rated as critical risk, and none were excluded from the analysis on this basis.

Detailed domain-level ratings are summarised in Table 3.

**Table 3:** ROBINS-I risk of bias assessment for the included studies ERAS: Enhanced Recovery After Surgery; NSQIP: National Surgical Quality Improvement Program; PS: propensity score; TKA: total knee arthroplasty; ASA: American Society of Anesthesiologists; BMI: body mass index; ROBINS-I: Risk of Bias in Non-randomised Studies of Interventions

Study	Bias due to confounding	Bias in selection of participants	Bias in classification of interventions	Bias due to deviations from intended intervention	Bias due to missing data	Bias in measurement of outcomes	Bias in selection of the reported result	Overall risk of bias
Matharu et al. [18]	Moderate: large registry analysis adjusted for multiple covariates, but no propensity matching; residual confounding inevitable	Low	Low	Low	Low	Low	Low	Moderate
Lee et al. [23]	Moderate: 1:1 propensity score matched, yet some unmeasured factors (e.g. ERAS use) not captured	Low	Low	Low	Low	Low	Low	Moderate
Pugely et al. [24]	Moderate: NSQIP cohort with multivariable and PS adjustment, but clinician choice of anaesthetic remains a source of bias	Low	Low	Low	Low	Low	Moderate	Moderate
Wei et al. [25]	Moderate: revision TKA cohort; PS weighting plus regression, but possible confounding by surgical complexity	Low	Low	Low	Low	Low	Low	Moderate
Park et al. [26]	Serious: single-centre; limited covariate control left ASA/BMI imbalance; depth of sedation differed	Low	Low	Moderate	Low	Low	Moderate	Serious
Walker et al. [27]	Moderate: 2:1 PS match, but administrative data lack granular comorbidities	Low	Low	Low	Low	Low	Low	Moderate
Stundner et al. [28]	Moderate: large Premier database; regression adjusted but hospital-level confounding likely	Low	Low	Low	Low	Low	Low	Moderate
Kendall et al. [29]	Moderate: ambulatory TKA cohort; 1:1 PS match, yet block/ERAS use not fully balanced	Low	Low	Low	Low	Low	Low	Moderate
Baldawi et al. [30]	Moderate: veteran population; 1:1 PS match, but residual disease severity confounding possible	Low	Low	Low	Low	Low	Low	Moderate

Results of the meta-analysis

Blood Transfusion Requirement

**Figure 2:**
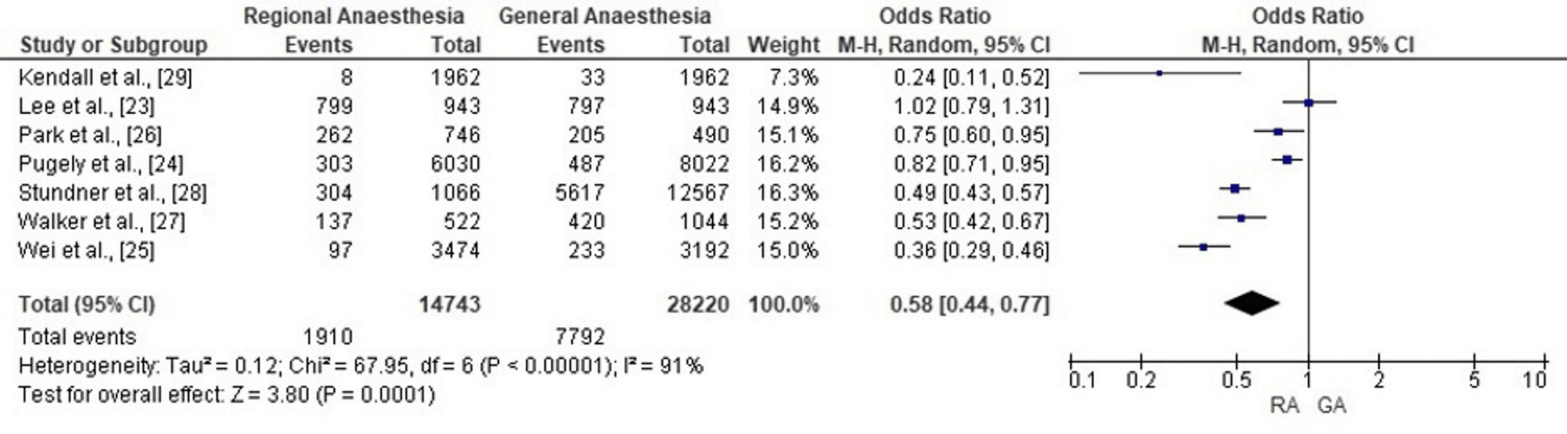
Forest plot of perioperative blood transfusion in total knee replacement under RA versus GA The pooled analysis demonstrates that RA is associated with a significantly reduced risk of requiring perioperative blood transfusion compared with GA in patients undergoing total knee replacement. RA: regional anaesthesia; GA: general anaesthesia Sources: [23-29]

Meta-analysis of seven cohort studies demonstrated that RA was associated with a significantly lower likelihood of perioperative allogeneic blood transfusion compared with GA (pooled OR: 0.58; 95% CI: 0.44-0.77; p<0.001). In absolute terms, transfusion rates ranged from approximately 6-12% with RA versus 10-20% with GA, representing an absolute reduction of 4-8 percentage points and a relative reduction of about one-third. Substantial statistical heterogeneity was observed (χ²=67.95; df=6; p<0.00001; I²=91%), but the direction of effect consistently favoured RA. The corresponding forest plot is presented in Figure 2.

Publication Bias Assessment for Blood Transfusion Outcome

**Figure 3:**
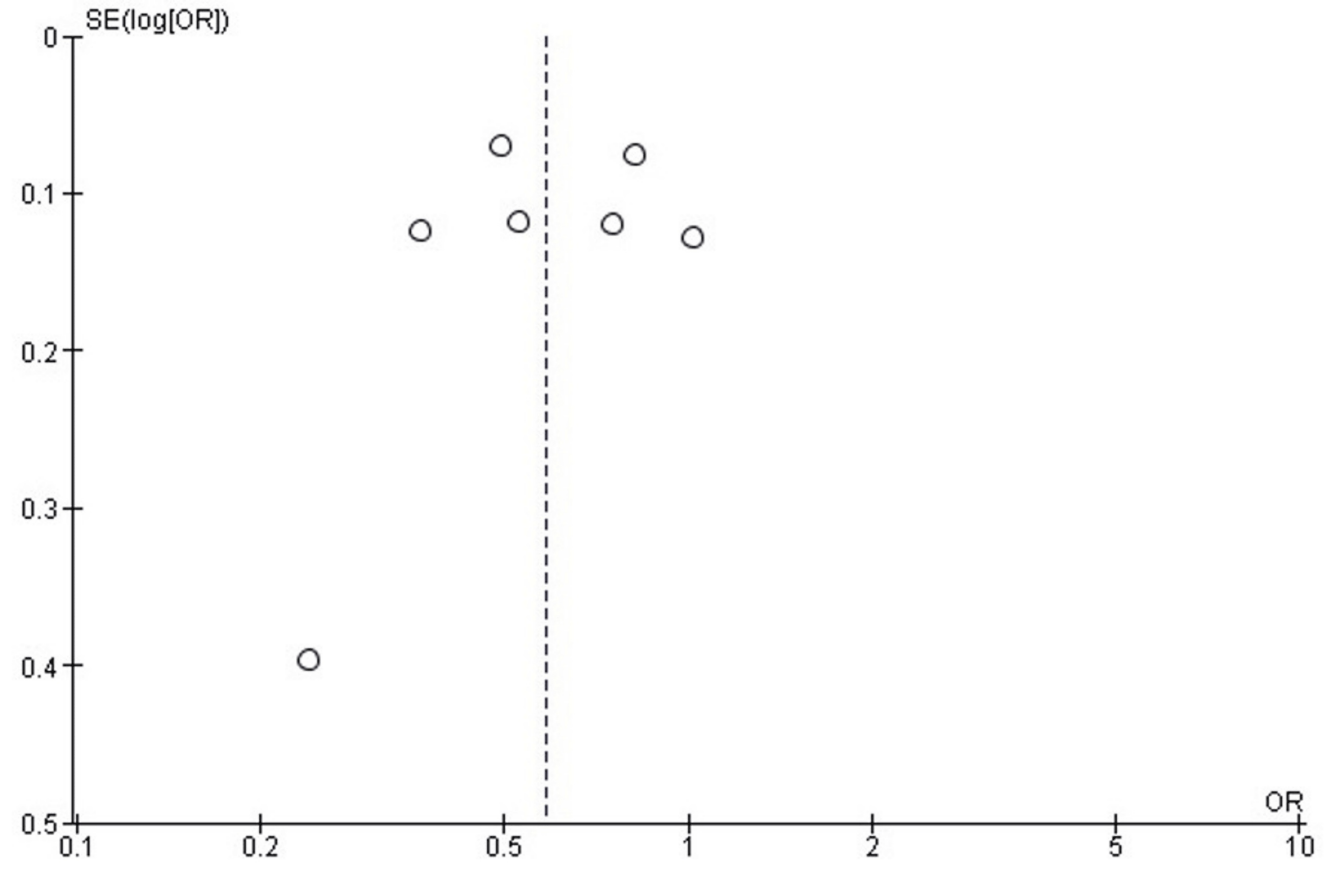
Funnel plot assessing the publication bias for perioperative blood transfusion outcome in total knee replacement Each dot represents an included study comparing RA with GA. The largely symmetrical distribution around the pooled odds ratio suggests the absence of publication bias. Egger's regression test showed no significant small study effect (p>0.05). RA: regional anaesthesia; GA: general anaesthesia

Visual inspection of the funnel plot revealed a largely symmetrical distribution of studies around the pooled odds ratio, suggesting the absence of publication bias. This observation was confirmed by Egger's regression test, which indicated no significant small study effect (p>0.05) (Figure 3).

Overall Complications

**Figure 4:**
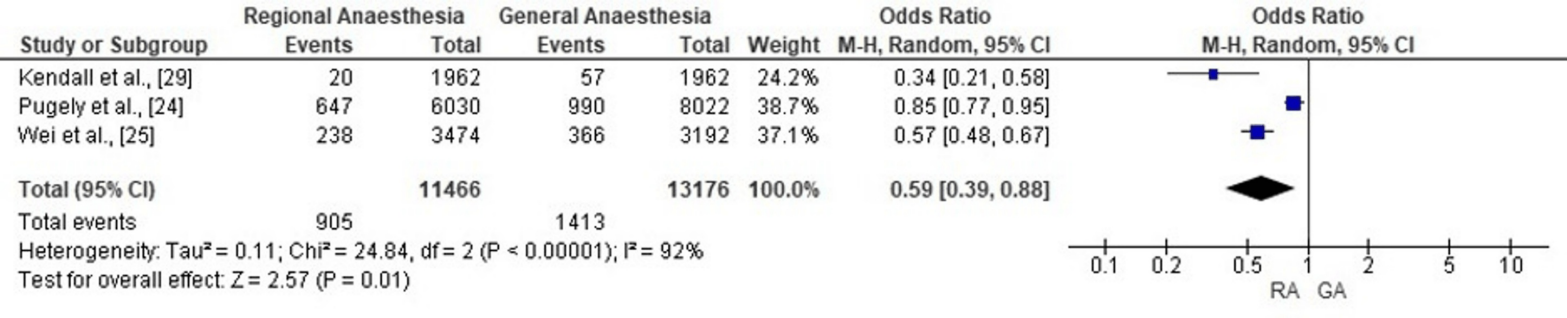
Forest plot comparing RA with GA for postoperative complications after total knee replacement The pooled odds ratio from three cohort studies indicates a significantly lower risk of 30-day postoperative complications with RA compared to GA. Error bars represent 95% CI. RA: regional anaesthesia; GA: general anaesthesia Sources: [24,25,29]

Meta-analysis of three cohort studies demonstrated that RA was associated with a 41% reduction in the odds of any 30-day postoperative complication compared with GA (pooled OR: 0.59; 95% CI: 0.39-0.88; p=0.01). Considerable heterogeneity was observed across studies (χ²=24.84; df=2; p<0.00001; I²=92%), indicating substantial variation in effect sizes. In absolute terms, complication rates ranged from 8-12% with RA versus 10-15% with GA, equating to approximately 2-4 fewer complications per 100 patients. The corresponding forest plot is shown in Figure 4.

Publication Bias Assessment for Overall Complications

**Figure 5:**
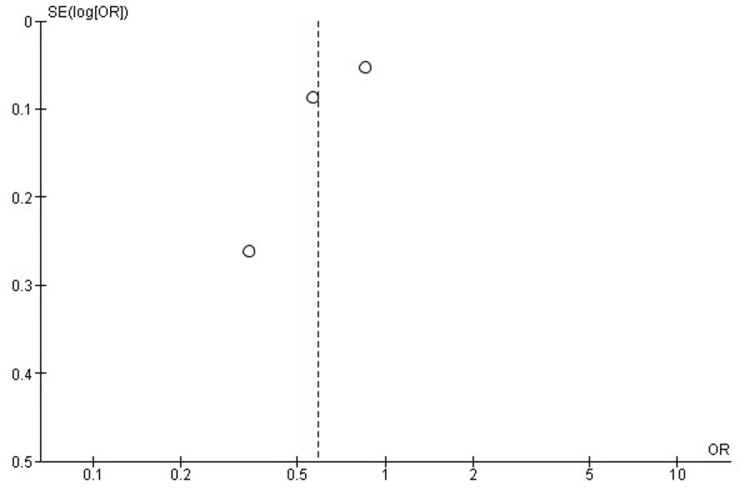
Funnel plot assessing the publication bias for postoperative complication risk in total knee replacement (RA vs. GA) Each point represents a study included in the meta-analysis comparing RA with GA. The approximately symmetrical distribution around the pooled effect suggests no obvious publication bias. Egger's regression test was non-significant (p>0.05). RA: regional anaesthesia; GA: general anaesthesia

Visual inspection of the funnel plot revealed an approximately symmetrical distribution of the three studies around the pooled effect, suggesting no apparent publication bias. Egger's regression test was non-significant (p>0.05); however, the small number of included studies limits the statistical power of this assessment (Figure 5).

Length of Stay

**Figure 6:**
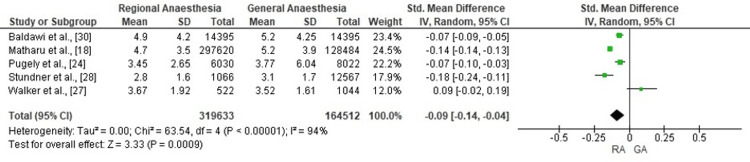
Forest plot comparing RA with GA for postoperative hospital length of stay after total knee replacement The pooled analysis from five cohort studies shows a small but statistically significant reduction in length of stay with RA compared to GA. SMD and 95% CI are shown for each study. RA: regional anaesthesia; GA: general anaesthesia; SMD: standardized mean difference Sources: [18,24,27,28,30]

Meta-analysis of five cohort studies demonstrated that RA was associated with a small but statistically significant reduction in postoperative hospital length of stay compared with GA (pooled SMD: -0.09; 95% CI: -0.14 to -0.04; p<0.001). In absolute terms, this corresponded to a mean reduction of approximately 0.3-0.5 days in hospital stay for patients receiving RA. Substantial heterogeneity was observed across studies (χ²=63.54; df=4; p<0.00001; I²=94%), although the direction of effect consistently favoured RA. The corresponding forest plot is presented in Figure 6.

Publication Bias Assessment for Length of Stay

**Figure 7:**
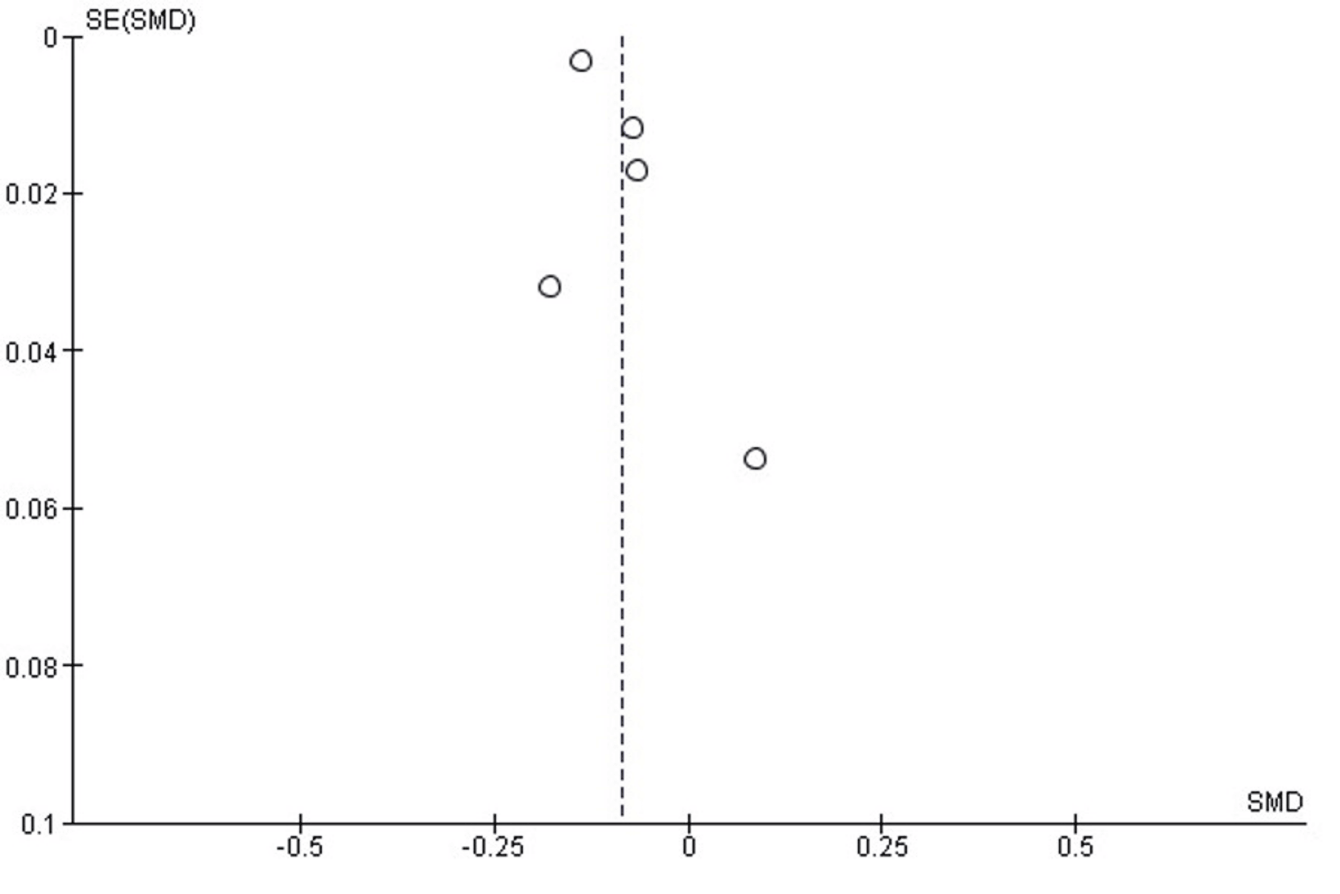
Funnel plot assessing the publication bias for length of stay differences between RA and GA in total knee replacement Each point represents a study included in the meta-analysis. The largely symmetrical distribution around the pooled effect suggests no obvious publication bias. Egger's regression test was non-significant (p>0.05), though the small number of studies limits statistical power. RA: regional anaesthesia; GA: general anaesthesia

Visual inspection of the funnel plot showed a largely symmetrical distribution around the pooled effect, suggesting no apparent publication bias. Egger's regression test did not detect a significant small study effect (p>0.05); however, the limited number of included studies reduces the statistical power of this assessment (Figure 7).

Discussion

The most consistent benefit observed was the reduction in perioperative blood transfusion with RA. Pooled analysis of seven studies demonstrated a 42% lower odds of transfusion in the RA group compared with GA (OR: 0.58; p=0.0001). This aligns with previous large-scale database studies, including Walker et al. [27] and Stundner et al. [28], where neuraxial techniques significantly reduced transfusion rates during bilateral TKR procedures.

The beneficial effects of RA on transfusion and complication rates are likely mediated by several physiological mechanisms. Neuraxial blockade induces sympathetic inhibition and controlled hypotension, reducing intraoperative blood loss from bone and soft tissues. Improved venous return and reduced central venous pressure further minimise bleeding from the highly vascular surfaces of the distal femur and proximal tibia. RA also attenuates the surgical stress response, lowering circulating catecholamines, cortisol, and inflammatory cytokines, which contribute to postoperative complications. By avoiding airway instrumentation and mechanical ventilation, RA preserves pulmonary function and reduces the risk of hypoxaemia and pneumonia. In addition, reduced exposure to volatile anaesthetic agents and opioids contributes to greater haemodynamic stability, lower incidences of thromboembolic and renal events, and earlier mobilisation. Collectively, these mechanisms explain the consistent reduction in transfusion requirements and perioperative complications observed across studies [27-30].

In addition, RA was associated with fewer postoperative complications. This effect may be explained by reduced exposure to systemic anaesthetic agents, attenuated stress hormone and inflammatory responses, and better preservation of pulmonary function compared with GA. By avoiding airway instrumentation and mechanical ventilation, RA decreases the risk of respiratory complications such as hypoxaemia and pneumonia. RA has also been linked to lower rates of thromboembolic events, potentially through earlier mobilisation and reduced hypercoagulability. Collectively, these physiological advantages may underpin the improved perioperative safety profile observed with RA.

The reduction in complication rates was another notable outcome. Meta-analysis of three studies showed a 41% reduction in overall 30-day complications in the RA group compared with GA (OR: 0.59; p=0.01). This benefit extended across multiple complication types, including cardiovascular, respiratory, renal, and infectious events. For example, Lee et al. [23] reported lower incidences of myocardial infarction and acute renal failure with neuraxial techniques, while Matharu et al. [18] and Kendall et al. [29] similarly found reduced risks of surgical site infection and urinary tract infection in RA cohorts. These findings suggest a broader protective effect of RA, potentially mediated by lower systemic stress responses, reduced opioid requirements, and faster mobilisation facilitated by motor-sparing blocks.

Hospital length of stay was modestly but significantly reduced with RA (SMD: -0.09; p=0.0009), with most studies reporting a 0.3-0.5-day advantage. This finding, observed by Matharu et al. [18], Park et al. [26], and Baldawi et al. [30], reflects the synergistic effects of enhanced recovery protocols and improved early postoperative outcomes with RA. Shorter length of stay may be explained by reduced opioid requirements, earlier mobilisation due to motor-sparing blocks, and integration with ERAS pathways. Shorter length of stay not only reduces healthcare costs but also lowers the risk of nosocomial complications and facilitates faster return to baseline function, particularly in ambulatory and high-volume surgical centres.

These benefits were consistent across varied TKR contexts. In ambulatory surgery, Kendall et al. [29] demonstrated that spinal anaesthesia reduced minor adverse events and transfusion rates without increasing readmission. In aseptic revision TKRs, Wei et al. [25] reported significantly lower odds of complications, transfusions, and prolonged hospital stays with RA. Even in simultaneous bilateral procedures, traditionally considered higher risk, RA was associated with fewer transfusions and a trend toward reduced morbidity [28].

Importantly, no study in this review reported disadvantages of RA compared with GA. Several authors explicitly recommended RA as the standard approach for TKR. Matharu et al. [18] advocated RA as the reference technique for both TKR and total hip replacement (THR), citing its favourable risk-benefit profile. Similarly, Park et al. [26] and Baldawi et al. [30] supported RA in high-risk and elderly patients due to its association with fewer systemic complications and shorter hospitalisation.

However, anaesthetic choice must remain individualised. While RA offers clear benefits, it may not be suitable for all patients. Factors such as spinal deformities, anticoagulant use, patient anxiety, or previous adverse experiences with neuraxial techniques may necessitate alternative approaches. A qualitative study by Bager et al. [22] highlighted the psychological distress some patients experience during spinal anaesthesia, underscoring the importance of patient education and shared decision-making in anaesthetic planning.

Across nine retrospective cohort studies encompassing 522,080 TKRs, RA consistently demonstrated superior short-term outcomes compared with GA. RA reduced the likelihood of perioperative blood transfusion by approximately one-third (6-12% vs. 10-20%), lowered the odds of any 30-day complication by about 40% (OR: 0.59; 95% CI: 0.39-0.88), and shortened hospital stay by roughly 0.3-0.5 days (SMD: -0.09; p<0.001). Despite substantial heterogeneity, the direction of effect uniformly favoured RA across all analyses. These conclusions are further supported by consistent quality ratings on both the ROBINS-I and Newcastle-Ottawa Scale (NOS) assessments [31], indicating generally moderate-to-high methodological quality across the included studies.

Limitations

A key limitation is that all included studies were retrospective cohorts; no RCTs were identified, limiting causal inference. All included studies were observational, introducing the potential for selection bias and residual confounding despite statistical adjustments. Follow-up periods were largely limited to short-term outcomes (mostly ≤90 days), restricting insights into long-term effects such as persistent pain or functional recovery. Additionally, high heterogeneity across studies likely reflects variations in surgical protocols, anaesthesia techniques, and patient characteristics. Finally, the limited number of studies for certain outcomes reduced the ability to fully assess publication bias.

## Conclusions

RA for TKR is associated with clinically and statistically significant advantages over GA, including ≈40% fewer perioperative complications, one-third lower transfusion rates, and a 0.3-0.5-day shorter hospital stay. These benefits were consistent across more than half a million procedures and support RA as the preferred anaesthetic technique, particularly for high-risk or elderly patients. Anaesthetic selection should remain individualised based on contraindications and patient preference. Future multicentre randomised trials are warranted to confirm these findings and to explore their impact on long-term function, cost-effectiveness, and patient satisfaction.
